# Laser-Induced Carbon Nanofibers as Permeable Nonenzymatic Sensor for Biomarker Detection in Breath Aerosol

**DOI:** 10.1021/acs.analchem.4c06580

**Published:** 2025-02-21

**Authors:** Selene Fiori, Christoph Bruckschlegel, Katharina Weiss, Keyu Su, Michael Foedlmeier, Flavio Della Pelle, Annalisa Scroccarello, Dario Compagnone, Antje J. Baeumner, Nongnoot Wongkaew

**Affiliations:** † Department of Bioscience and Technologies for Food, Agriculture and Environment, University of Teramo, Via R. Balzarini, 1, 64100 Teramo, TE, Italy; ‡ Institute for Analytical Chemistry, Chemo- and Biosensors, Faculty of Chemistry and Pharmacy, University of Regensburg, Universitaetsstrasse 31, 93053 Regensburg, Germany

## Abstract

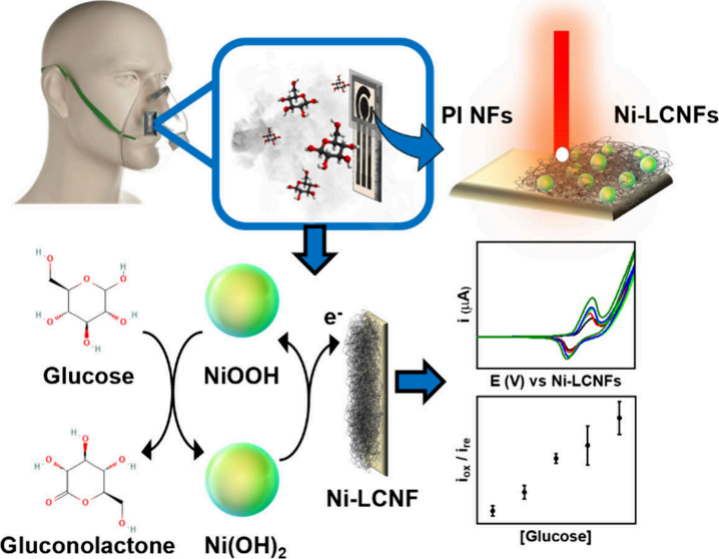

A novel breathable electrochemical enzyme-free sensor made from laser-induced carbon nanofibers embedding Ni nanocatalysts (Ni-LCNFs) is proposed for the capture and detection of biomarkers in breath aerosol. The permeable Ni-LCNF electrodes were fabricated on filter paper where a hydrophobic wax barrier was created to confine the device’s working area. The device was tested with aerosolized glucose, which was collected on the porous Ni-LCNF electrode. After a subsequent drying step, 0.1 M NaOH was dropped onto the device, and the electrocatalytic reaction of the captured glucose enabled by a Ni nanocatalyst was monitored via cyclic voltammetry (CV). Taking the oxidation/reduction peak ratios from CV as analytical signals improves the reliability and reproducibility of the glucose measurement. In the measurement step, closing the sensing area with adhesive tape, named *closed device*, enhances the detection sensitivity and enables the detection limit of 0.71 μM, which is 11.5 and 50 times, respectively, better when compared to the *open device* configuration. Measurements with simulated glucose aerosols containing clinically relevant glucose levels and comparison to screen-printed electrodes demonstrated the device’s superiority for breath analysis. Although *in vivo* validation studies must be conducted in future work, the proposed device results in a captivating point-of-care device integratable in breathing masks and breath analysis devices.

## Introduction

In the diagnostics field, the detection of biological biomarkers via noninvasive methods has received increasing attention, since they offer simple sampling and a more extensive screening capacity, avoiding injury due to the insertion of tools inside the body or skin puncture.^1,2^ Alternative to conventional biological fluids, i.e., saliva, urine, tears, sweat, plasma, blood, etc.,^2^ breath has become a captivating sample for noninvasive analysis. Indeed, breath can be easily collected in the form of aerosol and contains diagnostically important markers whose concentration is related to their content in blood.^3^ Nevertheless, the main problem with the analysis of exhaled breath aerosol is the concentration of the biomarkers, significantly lower than that of blood. Among others, glucose, a relevant biomarker for several diseases and chronic health problems, has an average concentration of 4.8 mM in blood in healthy people, while in respiratory fluids it is present in about 10-fold less amount.^4^ The glucose undergoes a further dilution during the condensation of exhaled breath aerosol, necessitating the use of a proper ‘breath collection’ strategy associated with adequate detection methods.^5^

Several analytical approaches are currently used for analysis of breath condensate such as gas chromatography, mass spectrometry, and laser spectroscopy.^1,6^ In this framework, electrochemical sensors are emerging as easy-to-use and low-cost analytical devices for clinical diagnostic.^7^ Electrochemical sensors include enzyme-based and enzyme-free detection strategies.^8^ The former has the advantage of high selectivity and sensitivity; nevertheless, it requires a dedicated enzyme immobilization process, needs controlled temperature, humidity, and pH, and often shows poor stability/storability.^9^ These drawbacks make enzyme-free sensors attractive, considering their facile preparation, low-cost, and suitability for mass production.^7,10^

Carbon nanofibers (CNFs) are useful sensing materials for electrochemical sensors, particularly for breath analysis. CNF films, properly manufactured/used, possess a 3D porous structure with high surface and electron-transfer capability.^11^ Noteworthy, thanks to their structure, CNF porous films enable effective permeation of gaseous phases, potentially allowing capturing/concentrating biomarkers in breath aerosol.^11^

CNFs are usually obtained via a two-step manufacturing process, including the production of a nanofiber precursor and consecutive carbonization. Several methods have been proposed for the production of nanofibers; among these, electrospinning turns out to be simple, versatile, and prone to large-scale production.^9,12^ Briefly, a polymer solution is subjected to a high voltage, and when the repulsive forces on the charged polymer exceed the surface tension of the formed droplet, a jet of the polymer is obtained,^13^ enabling nanofiber formation onto the collector. Subsequent carbonization is usually conducted at extremely high temperatures under inert gas.^9^ However, conventional carbonization approaches are not very suitable for direct electrode patterning and integration in sensors and devices, making them unfavorable for mass production.

On-site direct laser-induced fiber pyrolysis can effectively resolve the aforementioned issues. Indeed, CO_2_ laser treatment can straightforwardly convert electrospun polyimide (PI) nanofibers into laser-induced carbon nanofibers (LCNFs), allowing the formation of conductive porous films with on-demand shape and design.^14^ Moreover, simply adding a metal precursor into the PI spinning solution, LCNFs decorated with nanoparticles/nanostructures can be produced, improving the electron transfer ability and/or inducing additional catalytic features at the sensing film.^15^ This cosynthesis using the CO_2_ laser approach has proven to be efficient and practical for other materials such as graphene and conductive carbon inks where the as-developed sensors were successfully applied for H_2_O_2_, neurotransmitters and flavonoids determination.^16−20^

Our group has previously generated Ni-LCNF working electrodes demonstrating their ability in the nonenzymatic detection of glucose in basic environment.^21^ The Ni-LCNF electrodes were fabricated on a conductive indium tin oxide (ITO)-coated poly(ethylene terephthalate) (PET) support. In this pioneering work, even though the Ni-LCNF as a working electrode (WE) provided favorable analytical performance, the carbon nanofibrous sensing film does not provide permeability to gases, and the measurement setup necessitates the use of external reference (RE) and counter (CE) electrodes. These unfavorable features thus limit their applicability for breath sensor development. In another study by our group, we developed a manufacturing process where LCNF electrodes can be fabricated without a ITO/PET support.^22^ Herein, Fe-LCNF electrodes were generated and employed as WE, RE, and CE within a sensing device. The use of Fe-LCNFs as REs has proven to be reliable and stable for electrochemical measurements.

Current breath sampling strategies for analysis of exhaled breath require laborious and expensive instrumentation, and do not lend themselves well enough for further integration into point-of-care devices.^23^ The LCNFs are thus attractive to address these challenges. Furthermore, to our knowledge, electrodes with inherent porous structures coupled with a nonenzymatic detection strategy have not been yet investigated for both collecting and detecting analyte in breath aerosol.

In this work, a permeable Ni-LCNF-integrated sensor for breath analysis was developed, and its exploitability was proven by using aerosolized glucose as model analyte. The sensor allows a direct measurement of glucose from ‘the captured glucose aerosol’. The sensor design and catalytic features were optimized, together with the breath capture and measurement setup. Moreover, the effects of sensor storage conditions and interferent studies were also investigated. Eventually, we demonstrated the sensor’s capability to detect glucose at clinically relevant levels when integrated into a medical breathing mask.

## Experimental Section

### Sensor Manufacturing

**Figure 1:**
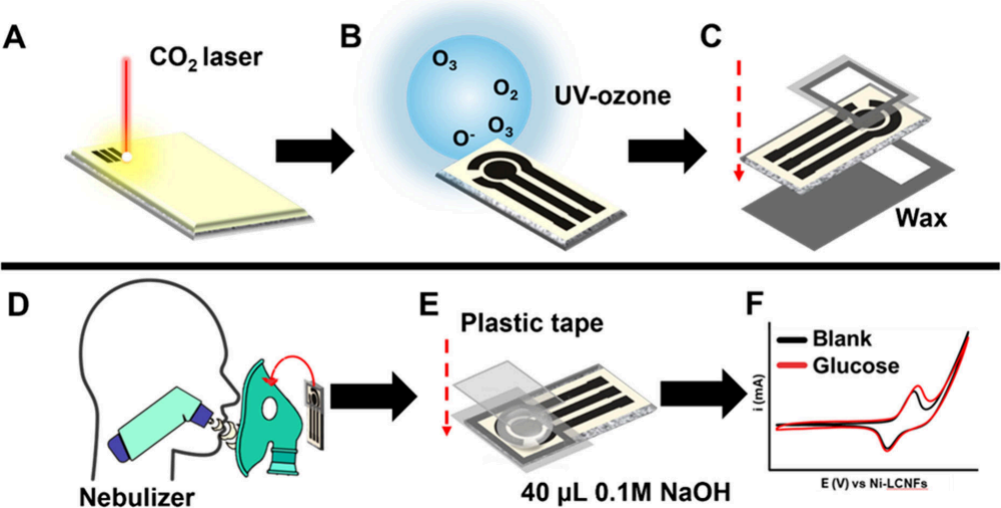
Schematic representation of sensor fabrication and measurement procedure. (A) Laser-induced carbon nanofibers (LCNFs) embedding Ni nanocatalyst generation using a CO_2_ laser. (B) UV-ozone treatment for enhancing the hydrophilicity of sensor’s surface. (C) Wax transfers to confine the sensor working area. (D) Experimental setup to test the potentiality of the sensor when integrated into a breathing mask. (E) Electrochemical measurement setup for determining the glucose aerosol after capturing in a closed configuration. (F) Example of cyclic voltammograms in the absence and presence of glucose.

In brief, electrospun PI nanofibers doped with Ni salt were fabricated on filter paper using a rotating drum collector (Figure S1). After the nanofibers were left to dry overnight under room conditions, they were subjected to a CO_2_ laser scribing process to obtain in one step laser-induced carbon nanofibers decorated with Ni-nanoparticles (Ni-LCNFs) (Figure 1A). For each nanofibers mat (size of 26 cm × 10 cm), 120 complete Ni-LCNF sensors are obtained; each sensor includes a working electrode (WE; d = 3 mm), reference electrode (RE), and counter electrode (CE), including the respective contacts (1 mm × 1 cm long). To improve the hydrophilicity of the sensing surface, sensors were treated with a UV-ozone cleaner for 5 min (Figure 1B). Afterward, to delimitate the sensor’s working area and insulate the back side, hydrophobic wax barriers were employed. The wax was initially printed on two plastic foils according to Figure 1C, and then, the sensor was sandwiched between them employing two glass slides held together by paper clips. Eventually, the wax was transferred into the sensor via melting in the oven (100 °C, 15 min), resulting in its transfer and percolation among nanofibers and paper pores (Figure 1C). Finally, the glass slides were disassembled, and the plastic foils were removed.

### Measurement Setup

Electrochemical measurements for glucose detection optimization were conducted by dropping 40 μL of the working solution onto the sensor working area; these measurements were performed without placing the plastic cover on top of the sensor, a configuration named *open device* (Figure 1C). Figure 1D illustrates the setup for measuring glucose in nebulized samples (breath simulation). To simulate the generation of breath aerosol, a plastic breathing mask with two lateral holes was placed onto a 3D-printed head model with an opening hole at the mouth, in which the nebulizer was positioned. The sensor was placed on the external side of the mask, positioning the sensor working area correspondingly to a mask lateral hole (the other hole was plugged) (Figure 1D). Aqueous glucose solutions were nebulized for 5 min, and the generated aerosol was collected on the sensor. After drying for 20 min at room temperature, 40 μL of 0.1 M NaOH was dropped on the working area of the sensor, and plastic tape was placed to close the working area (Figure 1E). This configuration, named *closed device*, forces the aerosol to penetrate the nanofibers, enhancing the detection sensitivity. Eventually, the measurement was performed via cyclic voltammetry (CV) in the potential range of window 0.0–1.0 V, using a scan rate of 50 mV s^–1^ and E step of 0.001 V; Ni-LCNF was used as WE, CE, and RE (Figure 1F).

For more details on experimental conditions, chemicals and materials, apparatus, and morphological characterization, please see the Supporting Information.

## Results and Discussion

### Sensor Fabrication and Measuring Glucose by Direct Detection Mode

Initially, to understand the ability of the Ni-LCNF sensors to directly determine glucose, cyclic voltammetry (CV) was run using the sensor in the configuration *open device* (please see also Figure S2 for detailed discussion on sensor fabrication and morphological structure of Ni-LCNFs). When CVs were performed in 0.1 M NaOH using three different Ni-LCNF sensors, high variations in current intensity and redox potential were observed (Figure S3) in which the detailed discussion and relevant reaction mechanism are given in the Supporting Information.

**Figure 2:**
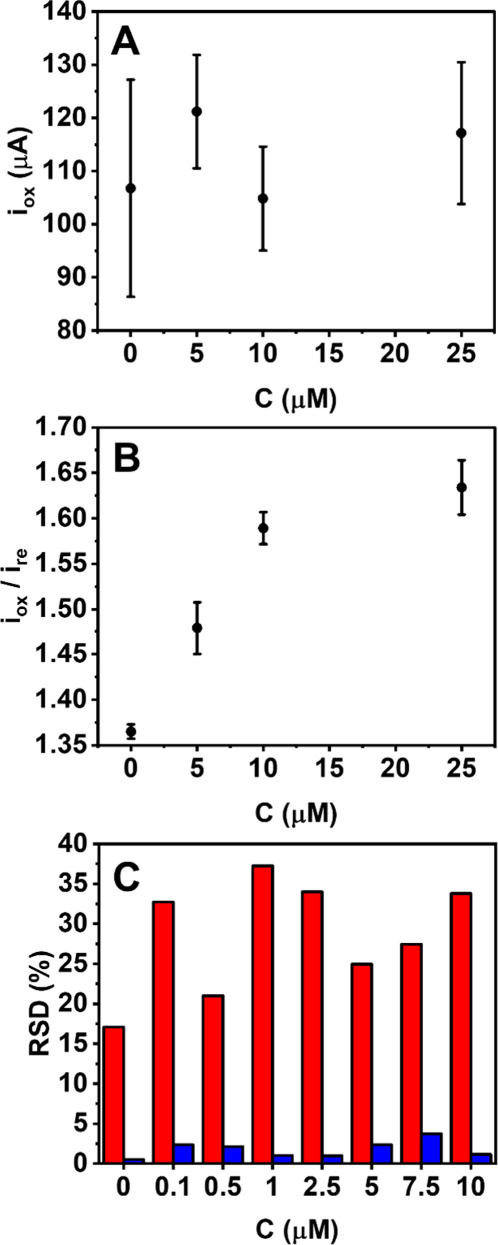
Evaluating the current signal from CVs. (A) Anodic peak current (i_ox_) obtained with glucose 5, 10, and 25 μM. (B) Peak intensity ratio (i_ox_/i_re_) with glucose 5, 10, and 25 μM. The data in A and B were extrapolated from the same CVs. (C) Comparison of relative standard deviation (RSD) calculated from anodic peak current (red) and i_ox_/i_re_ (blue) evaluated at concentrations of glucose enclosed between 0.1 and 10 μM. All data were obtained using the individual sensors (*open device* configuration) (*n* = 3).

The sensor was further tested in the presence of increasing concentrations of glucose; the obtained CVs are reported in Figure S4. In the presence of glucose, the redox behavior changes, and the oxidation reaction results in a more pronounced anodic peak. Unfortunately, the peak current obtained does not give rise to proper dose–response behavior, and the reproducibility of the signals is poor (RSD ≤ 31%, n = 3) (Figure 2A). This can be attributed to the high heterogeneity between devices (see also Figure S3), which makes absolute oxidation currents not reproducible. To overcome this issue, the ratio between the anodic and cathodic peak currents (I_ox_/I_re_) was employed to evaluate the glucose response. While the oxidation peak accounts for the active Ni(OH)_2_ sites on the electrode and the glucose concentration in the solution (see also reactions 1 and 3 in Supporting Information), the reduction peak well reflects the amount of as-generated NiO(OH) species in the electrocatalytic system (see also reaction 2 in Supporting Information) which can vary from electrode-to-electrode. Therefore, the cathodic peak current resulting from the NiO(OH) species can serve as an internal signal reference, indicating actual functional sites available on each electrode. As a result, this ratiometric data analysis facilitates the drastic improvement in signal reproducibility (Figure 2B and C). In addition, the analysis strategy greatly improves the dose response curve behavior, i.e., high correlation between signal and glucose concentration (Figure 2B vs A). This is attributed to proportionally decreased reduction peaks at higher glucose concentrations due to lower amounts of NiO(OH)_2_ during cathodic scans (see also the detailed explanation in Figure S4). Unfortunately, the reduction in cathodic peak intensity when increased glucose concentration cannot be seen clearly in the present study (Figure S4) owing to high variations between device-to-device. Nevertheless, this characteristic has been demonstrated in our pervious study where the same electrode was used to measure different glucose concentrations.^21^

In comparison to the widely used amperometric signal, the intrinsic self-correcting factors offered by the CV signal make our evaluation method more reliable, specifically for 3D-porous electrodes that possess high heterogeneity.^24−29^ In addition, unlike amperometric signals, the potential shifts from electrode-to-electrode that may occur do not significantly affect the ratiometric analytical signal.

### Sensor Electroanalytical Performance

Optimization of sensor production (Figure 1) was performed with respect to hydrophilic treatment and laser power to achieve the best analytical performance (*open device* configuration*)*. In brief, hydrophilic treatment via UV-ozone for 5 min yielded the Ni-LCNFs with high sensitivity and favorable reproducibility (Figure S5). Furthermore, the laser power was altered between 2.25 and 3.00 W (Figure S6), and 2.5 W was optimal, rendering the lowest limit of detection (LOD).

**Figure 3:**
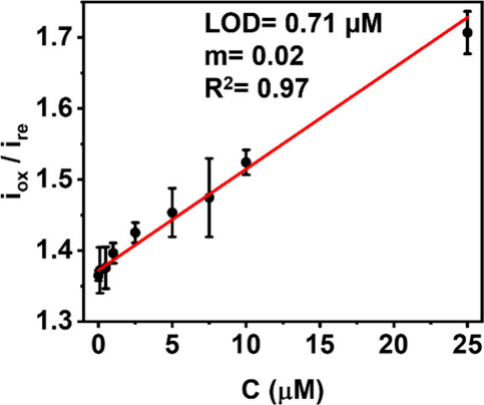
Calibration curve obtained with the *closed device* configuration (n = 3). Linear fit equation y = 0.0161[±0.0011]x + 1.3701[±0.0038], R^2^ = 0.9709. m = sensitivity (μM^–1^).

Previous experiments had shown that enclosing the LCNFs leads to improved sensor performance due to better, i.e., deeper and more homogeneous solution distribution. This approach is easily applicable by closing the working area with a plastic foil after the deposition of the working solution (Figure 1E). The *closed devices* rendered typical CVs as shown in Figure S7. As expected, the closed device remarkably enhanced the analytical performance as seen from the improvements in sensitivity (11.5 times) and LOD (50 times) in comparison to the *open device* (Figure 3 vs Figure S8). An LOD of 0.71 μM was obtained from 3 × S_*y*_/m (where S_*y*_ refers to the standard error of y-intercept, and m refers to the slope (or sensitivity), respectively), which together with the high hydrophilicity renders the closed devices indeed applicable for breath analysis. It should be noted that the LOD at the sub-μM range in this study can be achieved under stagnant conditions and is highly comparable to the previous reports where 3D-porous hybrid electrodes were amperometrically investigated for glucose sensing under stirring conditions (see also Table S1). This highlights the beneficial feature of 3D-porous electrodes within microelectroanalytical system in enhancing mass transport and their suitability for point-of-care (POC) testing.

### Capturing and Measurement of Aerosolized Glucose

The permeability to aerosols renders the carbon nanofiber electrodes highly attractive for capturing analytes present in the breath. Hence, breath analysis was simulated by integrating the sensor into a plastic breathing mask and placed on a 3D-printed head model; the measurement was performed in the *closed device* configuration after capturing (Figure 1E and F).

**Figure 4:**
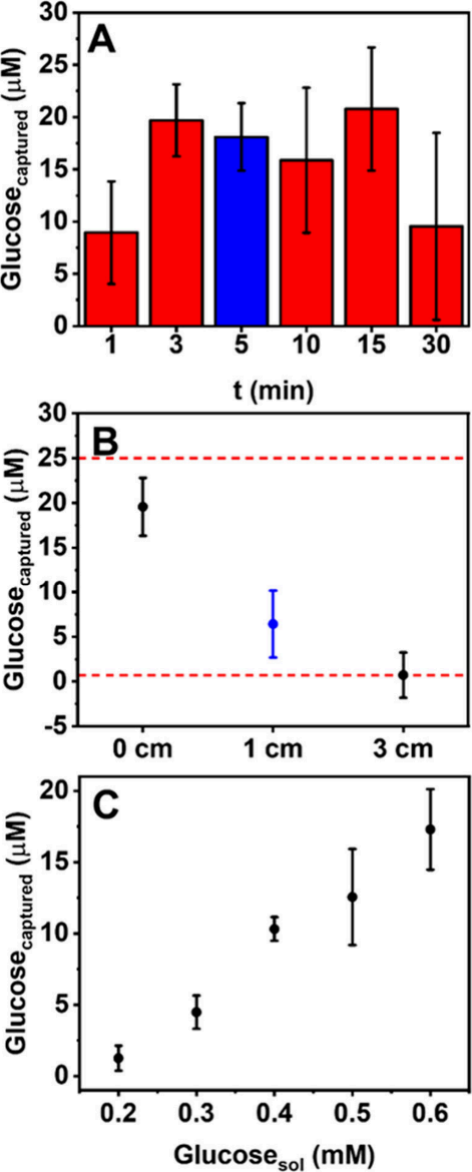
(A) The concentration of glucose measured with the *closed device* configuration after nebulization of 0.4 mM glucose solution at different times. (B) Optimization of the distance between the sensor surface and nebulization source. Graph reports the glucose concentrations obtained from nebulizing 0.4 mM glucose for 5 min, placing the sensor at 0, 1, and 3 cm of distance from the nebulizer. Red dashed lines represent the LOD and the superior limit of linear range. (C) Correlation between concentrations of glucose in the nebulized solutions and the captured glucose measured with the device (n = 3).

In initial studies performed without the head model, nebulized glucose was captured on the electrodes by placing them vertically over the nebulizer outlet. Aerosol collection time (Figure 4A) and the distance between the nebulizer outlet and the sensor (Figure 4B) were investigated.

The aerosol collection time was initially studied using a distance of 0 cm (meaning that the sensor was placed directly at the nebulizer outlet). A glucose concentration of 0.4 mM was selected for the nebulizer because this concentration mirrors the glucose level found in healthy lung fluid resulting from the typical ratio of glucose concentration in lung fluid to that in blood of approximately 1:12 (Figure 4A). The amount of glucose determined increased up to 3 min, while extending the collection time did not significantly increase glucose capturing ability. Instead, too long a collection time (30 min) negatively affects the measurement probably because the collected drops become larger and fall off the sensor. A collection time of 5 min was selected as the best compromise between the signal intensity and reproducibility. Nevertheless, as a nebulizer generates aerosolized glucose much more efficiently and intensely than the real breathing system, the proper collection time will have to be evaluated, especially also with respect to differences in breathing patterns of individuals.

Subsequently, the distance between the nebulization source and the sensor was investigated (Figure 4B). The distance of 0 cm was discarded since it is practically inapplicable in real situations. Therefore, as compromise, a practical distance of 1 cm (in blue in Figure 4B) was selected, since it can be used in real breath analysis allowing glucose level oscillation. Therefore, using the optimized parameters, the impact of the nebulizer solution concentration was studied, simulating physiological changes in lung fluid concentration. Glucose concentrations from 0.2 to 0.6 mM, corresponding to blood concentrations ranging from 2.4 mM to 7.2 mM (healthy individuals typically have about 5 mM glucose^4^), were studied (Figure S9).

As expected, an excellent correlation (r = 0.9761) between the glucose present in the nebulization solution and the sensor-captured glucose was obtained (Figure 4C); moreover, the sensor yields reproducible data (RSD ≤ 12%, n = 3).

In order to highlight the beneficial features of the proposed permeable sensors over traditional screen-printed carbon electrodes (SPEs) for capturing and detecting aerosolized glucose, their electrochemical performances were compared (Figure S10). A commercial SPE was modified via electrodeposition of a 10 mM solution of Ni(NO_3_)_2_ in 0.1 M KCl, carried out by CV in the potential range 0.0/–1.5 V for 3 consecutive scans.^30,31^ The presence of an oxidation peak at 0.55 V and a reduction peak at 0.15 V under alkaline conditions (Figure S10A), which are not present in the CVs of unmodified SPE (Figure S10A black lines and inset), confirms successful nickel deposition (note that the silver ink reference electrode shifts the peak potential compared to the Ni-LCNF reference electrode). Afterward, the Ni-modified SPE was tested to capture glucose. Surprisingly, the Ni-modified SPE allows glucose detection at 0.4 mM only when a drop of the solution is deposited onto the sensor surface, while it is unable to detect glucose from the nebulized solution (Figure S10). This result greatly emphasizes the advantages of the highly porous nanofiber-based sensors over conventional SPEs in capturing and detecting aerosolized analytes.

In the case of the sensor’s stability over time, it was demonstrated that shielding from air will be necessary for storage, because simply shielding from air via a closed container (Petri dish) vs unprotected ambient conditions resulted already in a dramatic increase in stability (Figure S11). Specifically, after 2 h, the signal from the unprotected sensor significantly decreased, compared to the freshly UV-ozone-treated electrode; subsequently, the sensor response further decreased over time. In contrast, shielding the sensors from the air, the sensor’s response is significantly less affected over time.

**Figure 5:**
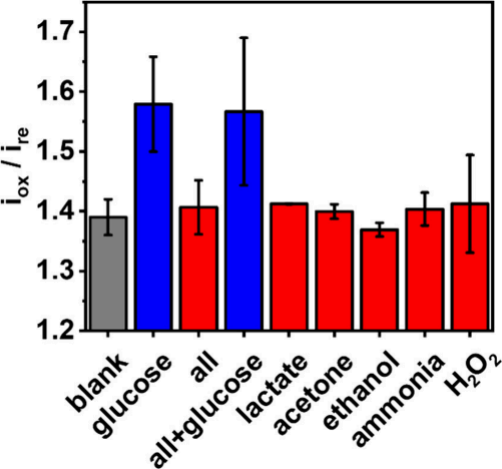
Selectivity study for potential interference found in breath. Glucose and interfering species at a concentration of 0.4 mM were used in nebulized solutions. The term “all” means a mixture of lactate, acetone, ethanol, ammonia, and H_2_O_2_.

Interferent studies were finally conducted. Overall, breath aerosol is considered a ‘clean’ sample matrix, majorly constituted of water. However, some potential interferent species, including lactate, acetone, ethanol, ammonia, and H_2_O_2_, can be copresent with glucose in the breath aerosol. Figure 5 greatly demonstrates the high selectivity of the proposed sensor toward glucose detection even in the presence of electroactive or potential interfering compounds present at the glucose-equimolar level. It was further attempted to investigate any interference from proteins by nebulizing diluted human serum (1:12). However, the employed nebulizer turned the test solution into foam which made reliable measurements impossible. Future interference studies with proteins will require a proper breath simulator setup. It may be also possible that the expected electrode fouling from large molecules may be tackled by ratiometric detection, which requires further investigation.

At this stage, it is foreseeable that the devices will be used for collection of breath aerosol and taken off of the patient before the electrochemical measurement. Therefore, there should be no risk from NaOH exposure for the patient. Furthermore, the combination of Ni with a noble metal, e.g., Pt or Au, within LCNFs could further allow the detection of glucose possible under physiological condition. Here, the noble metal can electrochemically create a basic environment (under a controlled cathodic voltage) for electrocatalytic reaction of glucose.^32^ As a result, online measurements could be achieved. The rapid advancement in software development nowadays can greatly facilitate the acquisition of data from CV measurements in a timely and reliable manner that suits the needs of POC testing.

## Conclusions

The current study highlights the potential of laser-induced carbon nanofiber (LCNF) electrodes and their breathability in directly sampling exhaled breath aerosol and detecting the analyte of interest. The devices are portable, low cost, and highly compatible with online sampling systems and operate at room temperature. In addition, by facilely dropping a reagent, NaOH in this case, onto the device after collection, the captured aerosolized analytes can be electrochemically detected by the device itself. As a result, this can reasonably reduce human error as no sample transfer is needed, unlike with exhaled breath condensate samples. Besides the electroactive nanocatalysts, other biorecognition elements, e.g., antibody and aptamers, combined with other electrochemical detection methods are feasible to incorporate into or onto LCNF electrodes. Therefore, the proposed devices can potentially accelerate the developments of breath analysis for other nonvolatile analytes, not limited to the glucose monitoring presented in this study. The analytical performance and practicality of the sensors for *in vivo* measurements will be investigated in the future work. These studies demand tight cooperation with clinicians in order to address all possible challenges and ensure maximum reliability prior to real-world applications.

## Data Availability

Data will be made available on request.

## Supporting Information Available

Experimental section consisting of chemicals and materials, apparatus, and morphological characterization. Results and discussion consisting of sensor fabrication and measuring glucose by direct detection mode, sensor electroanalytical performance, and capturing and measurement of aerosolized glucose. (PDF)
