# Supplementary material for: Laser-Induced Carbon Nanofibers as Permeable Nonenzymatic Sensor for Biomarker Detection in Breath Aerosol
Source: Anal Chem. 2025 Feb 21;97(8):4293–8. doi: 10.1021/acs.analchem.4c06580 (PMC11883729; doi:10.1021/acs.analchem.4c06580)
Supplement: Supplementary file 1 — ac4c06580_si_001.pdf [file ac4c06580_si_001.pdf]

SUPPORTING INFORMATION

Laser-Induced Carbon Nanofibers as Permeable Nonenzymatic Sensor for Biomarker Detection in Breath Aerosol

Selene Fiori[a,†], Christoph Bruckschlegel[b,†], Katharina Weiss[b], Keyu Su[b], Michael Foedlmeier[b], Flavio Della Pelle[a], Annalisa Scroccarello[a], Dario Compagnone[a], Antje J. Baeumner[b], Nongnoot Wongkaew[b, *].

[a] Department of Bioscience and Technologies for Food, Agriculture and Environment, University of Teramo, Via R. Balzarini, 1, 64100 Teramo TE, Italy

[b] Institute for Analytical Chemistry, Chemo- and Biosensors, Faculty of Chemistry and Pharmacy, University of Regensburg, Universitaetsstrasse 31, 93053 Regensburg, Germany

*Corresponding author

E-mail: nongnoot.wongkaew@ur.de

[†] S.F. and C.B. contributed equally to this paper

TABLE OF CONTENTS

	Page
**Experimental section:** ………………………………………………………..……..	S3
Chemicals and materials………………………………………………………………	S3
Apparatus……………………………………………………………………………………	S3
**Figure S1**. Fabrication of electrospun PI nanofibers doped with Ni (II) acetylacetonate…………………………………………………………………………	S4
Morphological characterization………………………………………………………	S4
**Results and discussion:** ……………………………………………………………	S5
Sensor fabrication and measuring glucose by direct detection mode………….....	S5
**Figure S2**. Sensor fabrication and morphological structure of Ni-LCNFs……………..	S5
**Figure S3.** High signal variations between devices……………………………………..	S6
**Figure S4.** Cyclic voltammograms obtained in presence of different amount of glucose…………………………………………………………………………..……	S7
Sensor electroanalytical performance………………………………………………	S8
**Figure S5.** Effect of UV-ozone treatment………………………………………………	S8
**Figure S6.** Influence of laser power on the limit of detection……………………...........	S9
**Figure S7.** Exemplary CVs obtained with increasing concentrations of glucose using the *closed device* configuration…………………………………………	S10
**Table S1.** Comparison of sensing response to other 3D-porous nonenzymatic glucose sensors…………………………………………………………………………..	S10
**Figure S8.** Dose response curve obtained from *open device* configuration…………	S11
Capturing and measurement of aerosolized glucose…………………………………	S12
**Figure S9.** Cyclic voltammograms obtained from different glucose concentrations nebulized………………………………………………………………	S12
**Figure 10.** Glucose measurement using Ni-modified commercial screen-printed electrodes………………………………………………………………………	S13
**Figure 11.** Stability of the sensor of the complete devices shown in Figure 1E………	S14
**References**……………………………………………………………………............	S14

Experimental section

Chemicals and materials. Solvent soluble PI Matrimid 5218 was purchased from Huntsman Corporation (The Woodlands, Texas, U.S.). Nickel (II) acetylacetonate, sodium hydroxide, D-glucose monohydrate, hydrogen peroxide, nickel (II) nitrate hexahydrate, potassium chloride, acetone (≥ 99.5% purity), ethanol (≥ 99.8% purity), ammonia and sodium L-lactate were purchased from Sigma Aldrich (St Louis, MO, USA). Dimethylacetamide (DMAc) was purchased from Acros Organics (Thermo Fisher Scientific Inc., Geel, Belgium). Screen-printed carbon electrodes were purchased from Metrohm DropSens (Llanera, Asturias, Spain). 100 mM stock solution of glucose was prepared in 0.1 M NaOH aqueous solution and stored at 4 °C.

Apparatus. Electrospinning was performed using a Starter Kit-Aligned 40kV (Linari Engineering srl, Pisa, Italy) with a rotating aluminum collector cylinder (diameter of 80 mm and length of 120 mm), equipped with a 5 mL glass syringe with a 20G needle. A CO_2 laser (VLS 2.30 system, 30W) from Universal Laser System Inc. (Scottsdale, Arizona, USA) with a 2.0'-lens in focus was used for laser scribing of nanofibers. To increase the hydrophilicity of Ni-LCNF film a UV-ozone cleaner from Jelight Co. Inc., model n° 42-220 (Irvine, California, USA) was employed, with a power of 200-240 V and frequency of 50 Hz. A wax printer (ColorQube 8880DN, Xerox Corporation, USA) was used to print hydrophobic regions onto polyester plastic foils (Overhead transparencies, thickness 0.10 mm, Avery Zweckform, Code: 3560) to define the working area and insulate the back of the sensors. Designs for the laser and wax printer were realized using CorelDRAW 2022 software. Electrochemical measurements were conducted using a multichannel potentiostat (MultiPalmSens4) from PalmSens (Palm Instruments BV, Houten, Netherlands). Breath simulation process was performed by nebulizing glucose solutions using an Inhalator from Medisana (Neuss, Germany), power 60 W, nebulization speed of 0.2 mL min^{-1}.

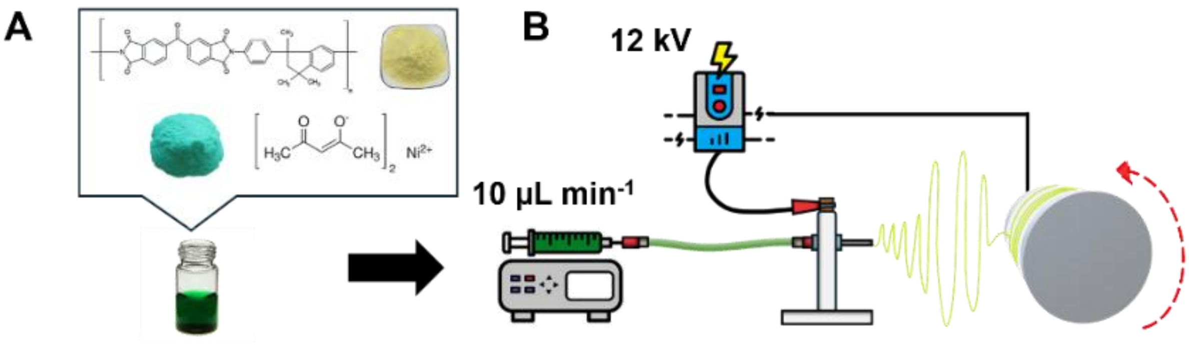

Figure S1. Fabrication of electrospun PI nanofibers doped with Ni (II) acetylacetonate. (A) Preparation of the spinning solution. **(B)** Electrospinning set-up to produce a nanofiber mat on filter paper.

In brief, a spinning solution containing 0.6 g of Matrimid and 0.15 g of Ni (II) acetylacetonate was prepared in a total volume of 4 mL of DMAc (**Figure S1A**). The solution was stirred overnight and subjected to the electrospinning process (**Figure S1B**). The electrospinning was conducted at a voltage of 12 kV with a flow rate of 10 µL min^{-1} for 5 h. The nanofibers were collected onto a chromatography filter paper (Whatman 1 CHR, 0.18 mm, 87 g m^{-2}) fixed around a drum rotating at 150 rpm. A tip-to-collector distance was maintained at 15 cm. The conditions of the spinning chamber were settled at 24 ± 1 °C and 34 ± 9 % of humidity.

CO_2 laser conditions employed: 2.55 W laser power, 76.2 cm s^{-1} laser speed 76.2 cm s^{-1}, and 1000 DPI (dots per inch) image density resolution.

Morphological characterization. Scanning electron microscope (Zeiss/LEO 1530, Germany) was used to characterize micro/nanostructure of the Ni-LCNFs film (**Figure S2I, i-iv)**; micrographs acquisition was done after Au-Pd sputtering. Sensor side-view micrograph (**Figure S2I-ii**) was obtained by cutting the sensor with the CO_2 laser in the cutting mode (laser power: 30W; laser speed: 50.8 cm s^{-1}).

Results and discussion

Sensor fabrication and measuring glucose by direct detection mode

Design of the sensors was based on a previously established sensor consisting of Fe-LCNFs as WE, RE, and CE. Furthermore, those had been made from a static nanofiber's collector generating 12 sensors per batch[1] which was here further advanced to collection on a larger rotating drum, allowing to obtain ca. 120 complete sensors per batch. The use of the rotating drum nanofibers collector produced strategically a nanofiber mat with random orientation (**Figure S2-iii**), with an average diameter of ca. 300 nm. The longer collection time per collecting area in this study 52 cm^2/h (vs. 32 cm^2/h)[1] made the fiber mat thicker (ca. 1.5-times) (**Figure S2-ii**), thus necessitates stronger laser power to sufficiently carbonize the entire nanofiber mat in vertical direction. Eventually, when subjected to laser treatment the nanofibers result in a conductive 3D-porous structure (**Figure S2-iv**).

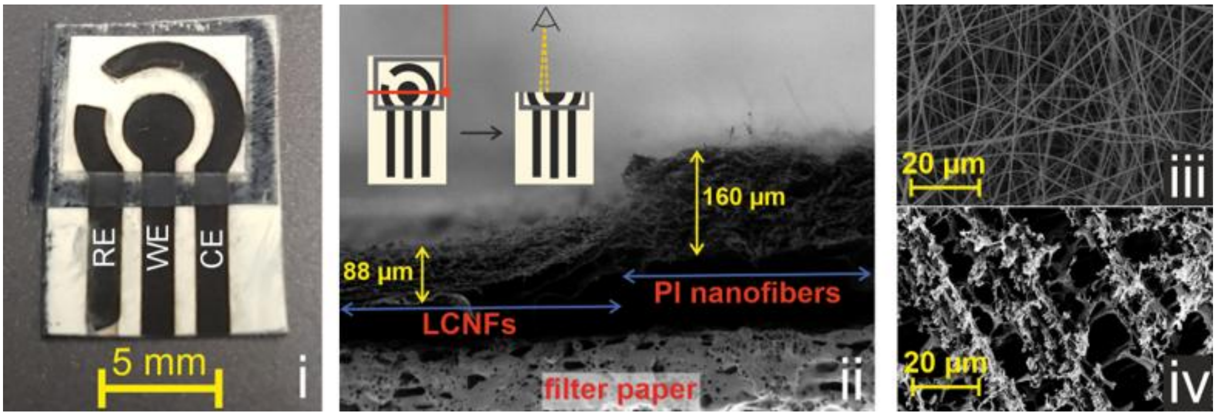

Figure S2. Sensor fabrication and morphological structure of Ni-LCNFs. Picture of the Ni-LCNFs device **(i)**. SEM micrograph of the sensor's side-view **(ii)**. Micrograph of the as-spun nanofibers **(iii)** and the Ni-LCNFs after device assembly **(iv)**.

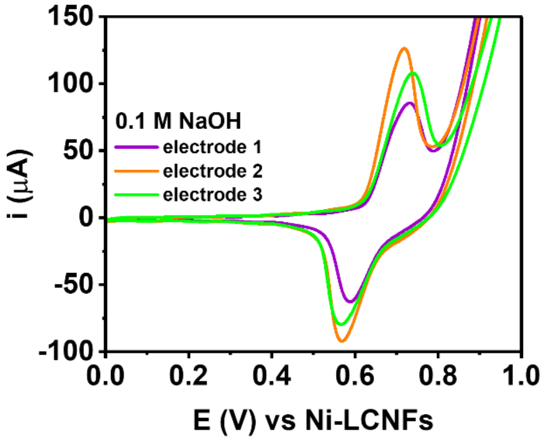

Figure S3. High signal variations between devices. The CVs obtained from three different Ni-LCNF sensors performed in 0.1 M NaOH without glucose.

Ni-LCNF sensors, in an alkaline environment, give rise to characteristic peaks; these peaks are attributable to the nano-Ni redox chemistry.[2,3] In particular, $Ni(OH)_2$ is oxidized during the anodic scan at around +0.7 V, following the reaction reported below.

$$Ni(OH)_2 + OH^- \rightarrow NiO(OH) + H_2O + e^- \qquad (1)$$

In the reverse scan (cathodic scan) the NiO(OH) is reduced at around +0.55 V, according to the reaction reported below.

$$NiO(OH) + H_2O + e^- \rightarrow Ni(OH)_2 + OH^- \qquad (2)$$

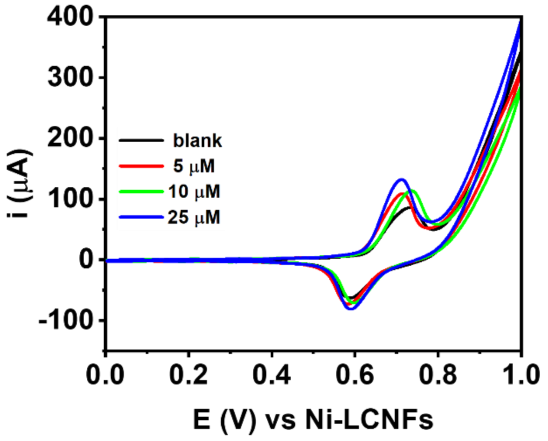

Figure S4. Cyclic voltammograms obtained in presence of different amount of glucose. CVs obtained from Ni-LCNF sensor performed in 0.1 M NaOH (blank) and with 5, 10 and 25 μM of glucose.

It is well known that, the NiO(OH) enables the electrocatalytic oxidation of glucose to gluconolactone following this reaction.[2,3]

NiO(OH) + glucose → $Ni(OH)_2$ + gluconolactone (3)

Therefore, the presence of glucose can lead to a concentration-proportional increase in oxidation currents, which can be commonly employed as analytical signals.

The signal responses and their oxidation potential shown in **Figure S4** (also in **Figure S7**) were random and might not well reflect the glucose levels under test. This issue is attributed to high surface heterogeneity between electrodes. Typically, when increasing glucose concentration the amount of $Ni(OH)_2$ also increases according to reaction 3, thus subsequently enhancing the anodic current at ca. +0.7 V as described in reaction 1. On the contrary, the amount NiO(OH) is lowered at higher glucose concentrations (reaction 3), thus the decrease in cathodic current (reaction 2) can be theoretically expected.

Sensor electroanalytical performance

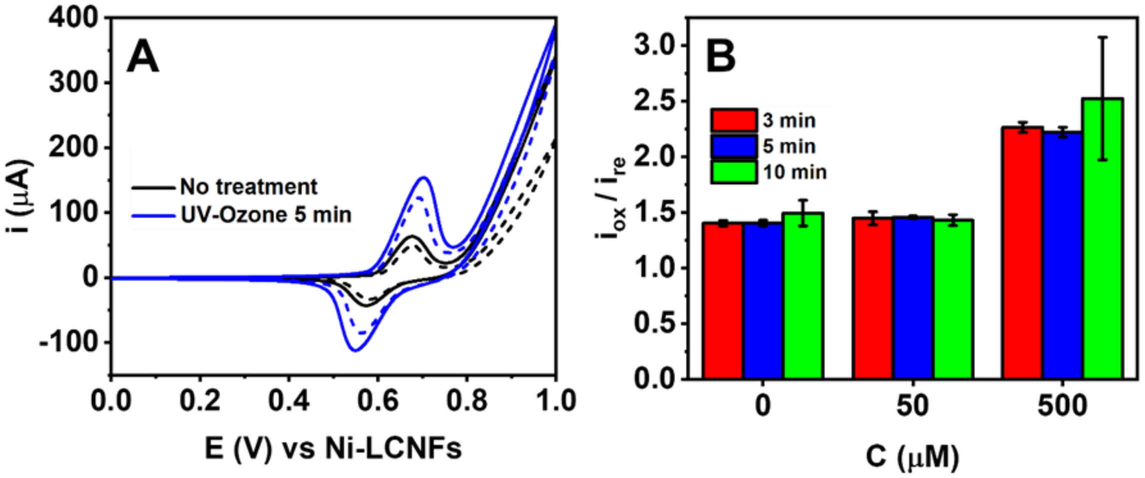

Figure S5. Effect of UV-ozone treatment. (A) Cyclic voltammograms obtained from Ni-LCNF sensor untreated and treated with UV-ozone (5 min, and 0.5 L min^{-1} O_2 flow rate). Measurements were performed in 40 µL of 0.1 M NaOH without (dashed lines) and with 50 µM glucose (continuous lines). **(B)** Comparison of signal ratio obtained in prensence of different amount of glucose, after UV-ozone treatment perfomed with different duration.

To achieve the best analytical performance, different parameters were optimized during the sensor fabrication process (cf. to **Figure 1** in the main manuscript). In this phase, the measurements were simply performed by dropping the NaOH solution containing glucose onto the working area (*open device* configuration).

The hydrophobicity of the pristine LCNF surface was hydrophilized to increase wettability via two different strategies, i.e., oxygen plasma treatment and UV-ozone treatment. Both techniques induce the formation of -OH and other oxygen-containing groups on the LCNFs surface, leading to higher hydrophilicity. The treatments greatly reduced the electrode variability, i.e. increasing the response reproducibility ($RSD_{non\text{-}treated} \leq 75\%$; $RSD_{plasma} \leq 22\%$; $RSD_{UV\text{-}Ozone} \leq 5\%$; n=3), with best effect provided by UV-ozone treatment. Interestingly, it did not significantly affect the LCNF referencing system, as can be seen from the slight peak potential shift in **Figure S5A** (approx. 30 mV - 25 mV vs. the untreated sensor). Further optimization (**Figure S5B**) demonstrated that 10 min-treatment was too aggressive and thus returned poor reproducible signals (RSD ≤ 22%, n=3). Even though treatment times of 3 and 5 min provided highly comparable performance, 5 min was chosen for further studies considering the greater signal reproducibility ($RSD_{3min} \leq 4\%$ vs. $RSD_{5min} \leq 2\%$, n=3).

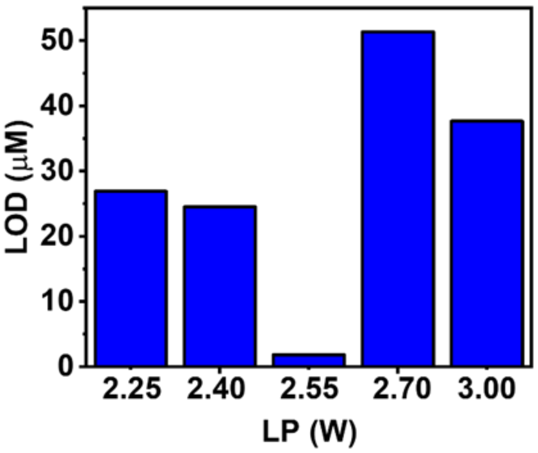

Figure S6. Influence of laser power on the limit of detection. Limit of detections calculated from the dose response curve (glucose concentration range 0-500 μM in 0.1 M NaOH). The measurements were performed using the *open device* configuration with triplicate measurement.

Also, the laser power (LP) used in the generation of Ni-LCNFs was altered between 2.25 and 3.00 W (**Figure S6**). The calculated limit of detection (LOD) tends to improve up to an LP of 2.55 W, after which it drastically worsened. It is assumed that excessive energy fluency destroys the nanofibers, whereas too little will not lead to full carbonization of the nanofibers and formation of nickel nanoparticles. According to the results, a laser power of 2.55 W was selected.

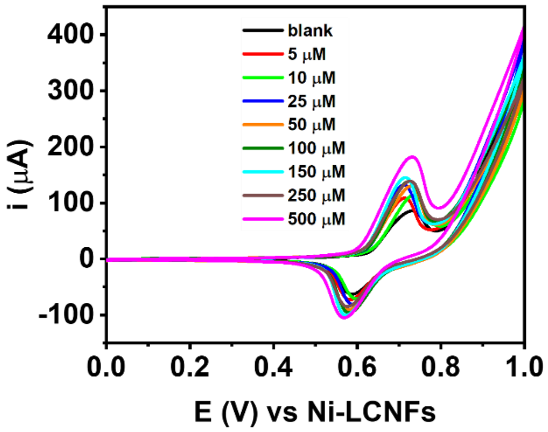

Figure S7. Exemplary CVs obtained with increasing concentrations of glucose using the *closed device* configuration.

Table S1. Comparison of sensing response in this work to other 3D-porous nonenzymatic glucose sensors

Electrodes	Electrochemical method	Measurement condition	LOD (μM)	Linear range (μM)
3D-porous NiO nanosheets vertically grown on graphite disk electrode[4]	Amperometry at 0. 5 V	0.5 M NaOH, stirred	0.9	up to 10000
3D porous Ni, N-codoped carbon material modified glassy carbon electrode[5]	Amperometry at 0. 5 V	0.1 M NaOH, stirred	0.15	1-1200
nickel nanoparticles-loaded 3D-porous magnetic graphene-like nanocomposite modified magnetic glassy carbon electrode[6]	Amperometry at 0. 5 V	0.1 M NaOH, stirred	1	2-220
3D porous $(Ni\text{-}Co)_3S_4$ nanosheets arrays on rGO-PEDOT hybrid modified glassy carbon electrode[7]	Amperometry at 0.6 V	0.05 M NaOH, stirred	0.503	1-5000
Copper hydroxide nanorods decorated porous graphene foam electrodes[8]	Amperometry at 0.6 V	1 M KOH, stirred	1.2	1.2-6000
Laser-induced carbon nanofiber embedded with Ni[2]	Amperometry at 0.55 V	0.5 M NaOH, stirred	0.3	10–100<br>100-5000
**This work**	**Cyclic voltammetry**	**0.1 M NaOH, non-stirred**	**0.71**	**up to 25**

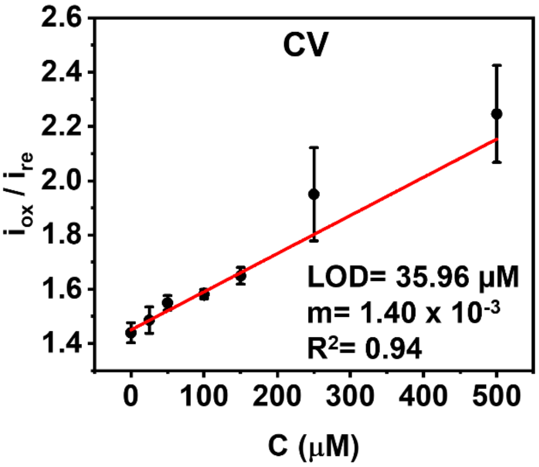

Figure S8. Dose response curve obtained from *open device* configuration. Linear fit of the data extrapolated from CV in the range 0-500 µM of glucose in 0.1 M NaOH. Equation: y=0.0014 [± 1.6422 x 10^{-4}] x + 1.4503 [± 0.0165], R^2= 0.9362 (n=3), m = sensitivity (μM^{-1}).

Capturing and measurement of aerosolized glucose

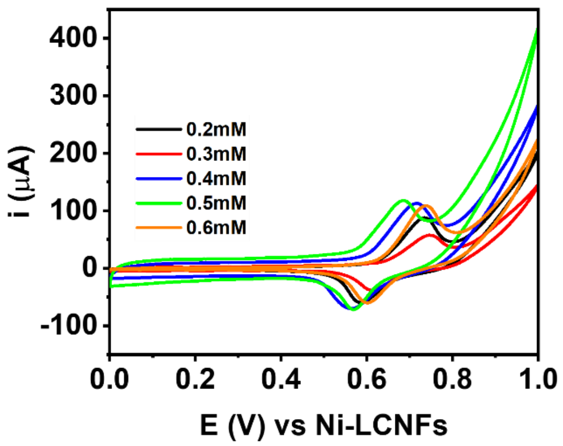

Figure S9. Cyclic voltammograms obtained from different glucose concentrations nebulized.

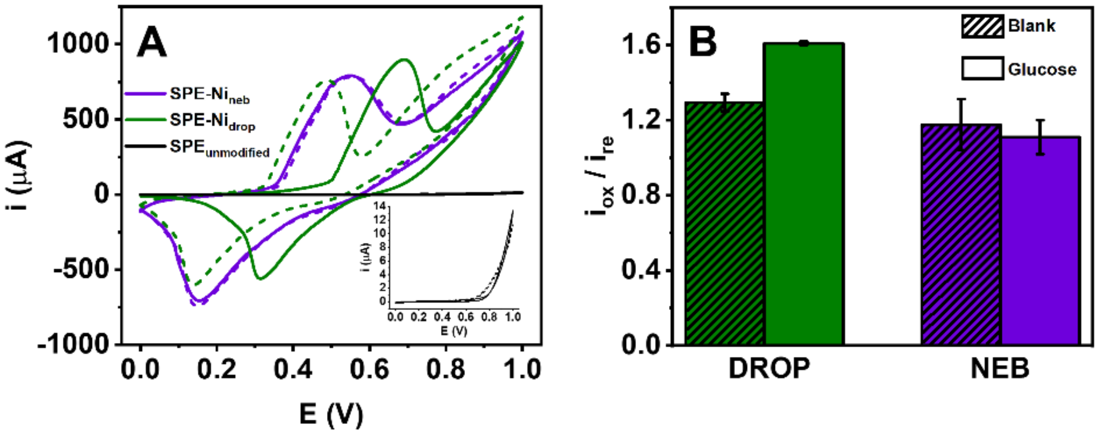

Figure S10. Glucose measurement using Ni-modified commercial screen-printed electrodes (A) Electrochemical behavior of SPEs with and without electrodeposited Ni. The inset reports the zoomed signal of the unmodified SPE. The CVs of SPE-Ni$_{drop}$ were obtained from drop-tesing for 0.1 M NaOH (dashed line) and 0.4 mM of glucose (solid line). The CVs of SPE-Ni$_{neb}$ were obtained after capturing nebulized glucose using 0 mM (dashed line) or O.4 mM (solid line). **(B)** Comparison of the I_{ox}/I_{re} with (0.4 mM glucose) and without glucose for drop and aerosol measurements using SPE-Ni electrodes.

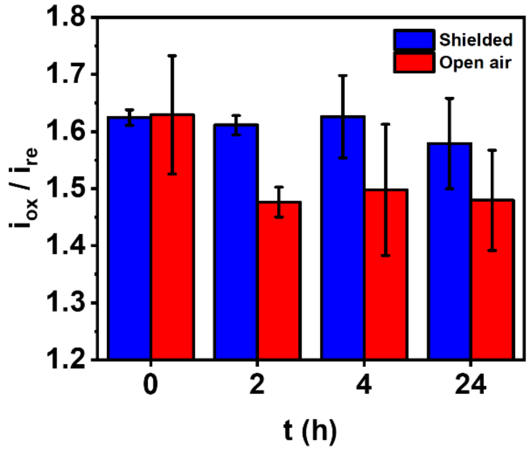

Figure S11. Stability of the sensor of the complete devices shown in Figure 1E (cf. to the main manuscript**).** Graph reports the electrochemical response from nebulized glucose solution at 0.4 mM.

References

(1) Perju, A.; Baeumner, A. J.; Wongkaew, N. Freestanding 3D-Interconnected Carbon Nanofibers as High-Performance Transducers in Miniaturized Electrochemical Sensors. *Microchimica Acta* **2022**, *189* (11), 1–13. https://doi.org/10.1007/s00604-022-05492-2.

(2) Simsek, M.; Hoecherl, K.; Schlosser, M.; Baeumner, A. J.; Wongkaew, N. Printable 3D Carbon Nanofiber Networks with Embedded Metal Nanocatalysts. *ACS Appl Mater Interfaces* **2020**, *12* (35), 39533–39540. https://doi.org/10.1021/acsami.0c08926.

(3) Franceschini, F.; Taurino, I. Nickel-Based Catalysts for Non-Enzymatic Electrochemical Sensing of Glucose: A Review. *Physics in Medicine* **2022**, *14*, 100054. https://doi.org/10.1016/j.phmed.2022.100054.

(4) Liu, H.; Wu, X.; Yang, B.; Li, Z.; Lei, L.; Zhang, X. Three-Dimensional Porous NiO Nanosheets Vertically Grown on Graphite Disks for Enhanced Performance Non-Enzymatic Glucose Sensor. *Electrochim Acta* **2015**, *174*, 745–752. https://doi.org/10.1016/j.electacta.2015.06.062.

(5) Chen, X.; He, X.; Gao, J.; Jiang, J.; Jiang, X.; Wu, C. Three-Dimensional Porous Ni, N-Codoped C Networks for Highly Sensitive and Selective Non-Enzymatic Glucose Sensing. *Sens Actuators B Chem* **2019**, *299* (July), 126945. https://doi.org/10.1016/j.snb.2019.126945.

(6) Wang, F.; Feng, Y.; He, S.; Wang, L.; Guo, M.; Cao, Y.; Wang, Y.; Yu, Y. Nickel Nanoparticles-Loaded Three-Dimensional Porous Magnetic Graphene-like Material for Non-Enzymatic Glucose Sensing. *Microchemical Journal* **2020**, *155* (February), 104748. https://doi.org/10.1016/j.microc.2020.104748.

(7) Meng, A.; Yuan, X.; Li, Z.; Zhao, K.; Sheng, L.; Li, Q. Direct Growth of 3D Porous (Ni-Co)3S4 Nanosheets Arrays on RGO-PEDOT Hybrid Film for High Performance Non-Enzymatic Glucose Sensing. *Sens Actuators B Chem* **2019**, *291* (March), 9–16. https://doi.org/10.1016/j.snb.2019.04.042.

(8) Shackery, I.; Patil, U.; Pezeshki, A.; Shinde, N. M.; Kang, S.; Im, S.; Jun, S. C. Copper Hydroxide Nanorods Decorated Porous Graphene Foam Electrodes for Non-Enzymatic Glucose Sensing. *Electrochim Acta* **2016**, *191*, 954–961. https://doi.org/10.1016/j.electacta.2016.01.047.
